# *Fusobacterium*-associated molecular and immunological alterations in colorectal cancer: Insights from a Norwegian cohort

**DOI:** 10.3389/fimmu.2025.1601423

**Published:** 2025-08-14

**Authors:** Thura Akrem Omran, Jose Luis Subirats Camacho, Thulasika Senthakumaran, Gro Gundersen, Annette Knapskog Alte, Ulla Randen, Hege Smith Tunsjø, Per Christian Sæther, Vahid Bemanian

**Affiliations:** ^1^ Department of Life Sciences and Health, Oslo Metropolitan University, Oslo, Norway; ^2^ Department of Pathology, Akershus University Hospital, Lørenskog, Norway; ^3^ Faculty of Medicine, University of Oslo, Oslo, Norway; ^4^ Department of Microbiology and Infection Control, Akershus University Hospital, Lørenskog, Norway; ^5^ Department of Immunology and Transfusion Medicine, Akershus University Hospital, Lørenskog, Norway

**Keywords:** colorectal cancer (CRC), gene expression, immune genes, *Fusobacterium*, fap2, Norway

## Abstract

**Background:**

The gut microbiome may significantly influence the development of colorectal cancer (CRC), with *Fusobacterium* species playing a key role. Recent research has identified *Fusobacterium animalis* as the predominant *Fusobacterium* species in CRC tumors. This pilot research explores the immunological and molecular interactions associated with *F. animalis* and other Fusobacterium species in Norwegian CRC patients.

**Methods:**

Tumor samples from 25 CRC patients were divided by *Fusobacterium* load and analyzed for molecular alterations, immunological gene expression, and macrophage polarization. *Fusobacterium*-high tumors were associated with microsatellite instability (MSI).

**Results:**

Analysis of differential immune gene expression, combined with correlation analyses, identified 25 genes, including C-X-C motif chemokine ligand 8 (CXCL8), interleukin-6 (IL6), indoleamine 2,3-dioxygenase 1 (IDO1), and secreted phosphoprotein 1 (SPP1), that exhibited significant associations with *Fusobacterium* abundance in this cohort. Analysis of *Fusobacterium* adhesion protein 2 (Fap2) revealed active transcription and constitutive expression across multiple colonic sites, including CRC tumor tissues, adjacent non-neoplastic tissues, the ascending colon, and the sigmoid colon. The analysis revealed a positive correlation between RNA levels of Fusobacterium-specific genes (fap2 and nusG) and immune genes (CXCL8, IL6, SPP1, and IDO1) across different colonic sites. This suggests that the abundance of active *Fusobacterium* cells is related to and possibly influences the pro-inflammatory response in the colonic microenvironment. Although arginase 1 (ARG1) expression was elevated in Fusobacterium-high tumors, no significant link was found between Fusobacterium abundance and M2 macrophage polarization, contradicting previous studies.

**Conclusions:**

High *Fusobacterium*, dominated by *F. animalis*, was linked to increased immune gene expression and constitutive fap2 activity. M2 polarization was unaffected, possibly reflecting in vivo tumor complexity.

## 1 Introduction

Over the past two decades, research has demonstrated a potential role of the gut microbiome in the development and progression of colorectal cancer (CRC) (1). Among the microbial taxa implicated in CRC, the genus *Fusobacterium* has emerged as a key player (2). *Fusobacterium* is a Gram-negative, obligate anaerobic bacterium commonly located in the oral cavity. For over a decade, it has been studied for its role as an onco-microbe. Already in 2012, Kostic et al. reported increased levels of *F. nucleatum* in CRC tumors (3). Ever since, there have been accumulating studies linking *Fusobacterium* to the development and progression of CRC (4).

*F. nucleatum* was initially classified as a single species comprising several subspecies*: F. nucleatum* ssp. *nucleatum*, *F. nucleatum* ssp. *animalis*, *F. nucleatum* ssp. *vincentii*, and *F. nucleatum* ssp. *polymorphum* (5). However, advancements in genomic studies have shown significant divergence across these subspecies, leading to the elevation of *F*. *animalis* and *F. vincentii* to species designations (5, 6). *F. animalis* has emerged as the predominant species detected in CRC tumors (7–9). In contrast, *Fusobacterium* species associated with other diseases, such as periodontitis, brain abscesses, and adverse pregnancy outcomes, are primarily *F. nucleatum* (2). This distinction indicates that *F. animalis* is specifically adapted to the CRC tumor environment. Other species, such as *F. nucleatum, F. necrophorum*, and *F. periodonticum*, are also reliably found in lesser proportions among CRC patients (7–9). As a result, a virulent genus-level *Fusobacterium* CRC complex, likely including multiple *Fusobacterium* species, has been proposed, redirecting focus from rigid taxonomic classification (10, 11).

*Fusobacterium* species have several putative virulence factors, including adhesion proteins (FadA), the arginine-inhibitable adhesin RadD, and the galactose-inhibitable adhesion protein 2 (Fap2), which may contribute to CRC progression at different stages (12–14). In recent years, there has been increasing focus on the Fap2 protein and its role in colorectal carcinogenesis, and *F. nucleatum* Fap2 has been identified as a key factor in the invasion of cultured cancer cells and the subsequent secretion of pro-inflammatory cytokines (15). Fap2 is a type 5 secretion system (T5SS) autotransporter that mediates *Fusobacterium* to interact with its environment, particularly host cells. Fap2 has a hook-like structure that binds to the lectin N-acetylgalactosamine (Gal-GalNac) that is highly expressed on the surface of cancer cells and to the T cell immunoreceptor with Ig and ITIM domains (TIGIT) present on the T cells and natural killer cells (13, 16). Additionally, Fap2, acting as an autotransporter, facilitates the export of FadA to the bacterial surface, where it contributes to adhesion and invasion of host cells (17). Yeoh et al. identified 754 putative *fap2* homologs in 288 *Fusobacterium* genomes and demonstrated that *fap2* homologs are widely dispersed in *Fusobacterium* genomes, although with significant sequence diversity (11). It is not yet known whether the diversity in the *fap2* sequence results in different Fap2 binding abilities to cancer cells or if Fap2 is more frequently present in *F. animalis* compared to other *Fusobacterium* species. Some studies have proposed that clinical strains of *Fusobacterium* naturally lacking *fap2* show reduced binding to Gal-GalNac-expressing CRC cells (13). However, expression studies of the *fap2* gene are limited, and it remains unclear whether *fap2* expression is increased in the tumor microenvironment.

Studies link *Fusobacterium* to oncogenic mutations, epigenetic changes, and microsatellite instability (MSI-H) in CRC (12, 18, 19). However, it remains unclear whether *Fusobacterium* drives these changes or simply thrives in the evolving tumor microenvironment. Additionally, *Fusobacterium* has been demonstrated to enhance a pro-inflammatory tumor microenvironment (TME) through its interactions with immune cells and the induction of inflammatory cytokine secretion. This response may facilitate tumor growth, invasion, and immune evasion (18, 20, 21). An increasing number of studies have shown that the binding and invasion of cancer cells by *Fusobacterium* species stimulate the secretion of pro-inflammatory cytokines such as tumor necrosis factor (TNF), interleukin-6 (IL6), interleukin-1 beta (IL1β), and C-X-C motif chemokine ligand 8 (CXCL8 or IL8). Casasanta et al. reported an Fap2-dependent invasion of *F. nucleatum* in an HCT116 cell line, resulting in the secretion of CXCL8 and C-X-C motif chemokine ligand 1 (CXCL1), thereby enhancing the metastatic potential of the cancer cells (15). Previous analyses of the present cohort revealed significantly elevated immune gene expression (*CXCL1*, *IL1B*, *IL6*, *CXCL8*) in most CRC tumors (22). Cell culture studies conducted by Udayasuran et al. showed that *CXCL8* and *CXCL1* gene expression is induced under hypoxic conditions independently of *Fusobacterium* infection, with hypoxia promoting *Fusobacterium* invasion and exacerbating the hypoxia-induced effects (21). However, the *in vivo* correlation between increased *CXCL8*/*CXCL1* expression and *Fusobacterium* presence remains insufficiently explored. Moreover, the majority of *in vitro* studies have utilized single strains of *Fusobacterium*, predominantly *F. nucleatum*, thereby leaving a gap in our understanding of the impact of different *Fusobacterium* species, in particular *F. animalis*.

In addition to its role in promoting metastasis, CXCL8 has been shown to induce an immunosuppressive TME by promoting M2 macrophages and inhibiting CD8+ T cell infiltration (23). Tumor-associated macrophages (TAMs) can polarize into M1 (pro-inflammatory) or M2 (anti-inflammatory) phenotypes, influencing tumor progression, metastasis, and prognosis (24). In both colorectal and oral cancer, *Fusobacterium* has been shown to impair TAMs function, which plays a significant role in tumor progression (25). Studies conducted *in vitro* and *in situ* demonstrated that *F. nucleatum* drives M2 macrophage polarization via the IL6/Signal Transducer and Activator of Transcription 3 (STAT3)/c-Myc signaling pathway and the Toll-like receptor 4 (TLR4)/Nuclear factor kappa B (NF-κB)/S100 calcium-binding protein A9 (S100A9) cascade (26, 27). M2 macrophages are characterized by the production of anti-inflammatory and pro-tumorigenic factors such as interleukin-10 (IL10), transforming growth factor beta (TGFβ), and arginase-1 (ARG1). These factors suppress anti-tumor immune responses, degrade the extracellular matrix, and promote cancer cell migration and invasion, creating a supportive TME (27). Additionally, the M2 polarization helps tumors evade immune surveillance and drives CRC metastasis (24). However, it is important to emphasize that *in vitro* studies may not fully elucidate the complex interactions among various cell types within the colorectal cancer tumor environment.

We have previously characterized the immune and bacterial profiles in colorectal tumors from a small cohort of Norwegian patients. Our results indicated a profound inflammatory response in the CRC tumors and defined a distinctive immunological signature (22). Furthermore, our microbiome analysis demonstrated a high prevalence of *Fusobacterium* in these tumor biopsies and identified the *Fusobacteria* to the species level (8, 28, 29). The integration of these data may provide valuable insights into the association of *Fusobacterium* and immunological gene expression in CRC. The aim of this study was to examine the link between immune gene expression in CRC tumors, *Fusobacterium* species, their virulence factor Fap2, and M2 macrophage polarization.

## 2 Materials and methods

### 2.1 Study cohort and sample collection

The study group consisted of 25 CRC patients who were scheduled for colonoscopy at Akershus University Hospital (Ahus) from 2014 to 2017. Colonoscopy examinations were conducted for several reasons, such as gastrointestinal bleeding, weight loss, alterations in bowel habits for more than four weeks, or the detection of colorectal abnormalities during computed tomography (CT) colonography. All biopsy samples were gathered during the first colonoscopy, before the diagnosis was made and prior to starting any treatment for CRC. Biopsies of the colonic mucosa, measuring 2–3 mm, were collected from individuals with a cancer diagnosis at four sites: the ascending colon (AC), the cancerous tissue (TU), the non-neoplastic tissue (NN) nearby the cancer (located 5 cm afar), and the sigmoid colon (CS). **Table 1** contains comprehensive information about the patients and samples. The biopsies were stored in Allprotect Tissue Reagent (Qiagen, Hilden, Germany) according to the manufacturer’s guidelines. The extraction of DNA and RNA was carried out as previously described, following the method outlined by Moen et al. with modifications made to the AllPrep DNA/RNA mini kit from Qiagen (22, 30).

**Table 1:** Characteristics of the study group.

Characteristics	Number of patients (n = 25)
Age (mean/median)	69.3 years
Sex distribution: n (%)	
- Male	18 (72%)
- Female	7 (28%)
Tumor location: n (%)	
- Cecum	6 (24%)
- Ascending colon	3 (12%)
- Transverse colon	2 (8%)
- Sigmoid colon	11 (44%)
- Rectum	3 (12%)
Tumor stage (Dukes): n (%)	
- A/B	14 (56%)
- C	6 (24%)
- D	4 (16%)
- Unknown	1 (4%)

### 2.2 *Fusobacterium* abundance in tumor samples

**Figure 1:**
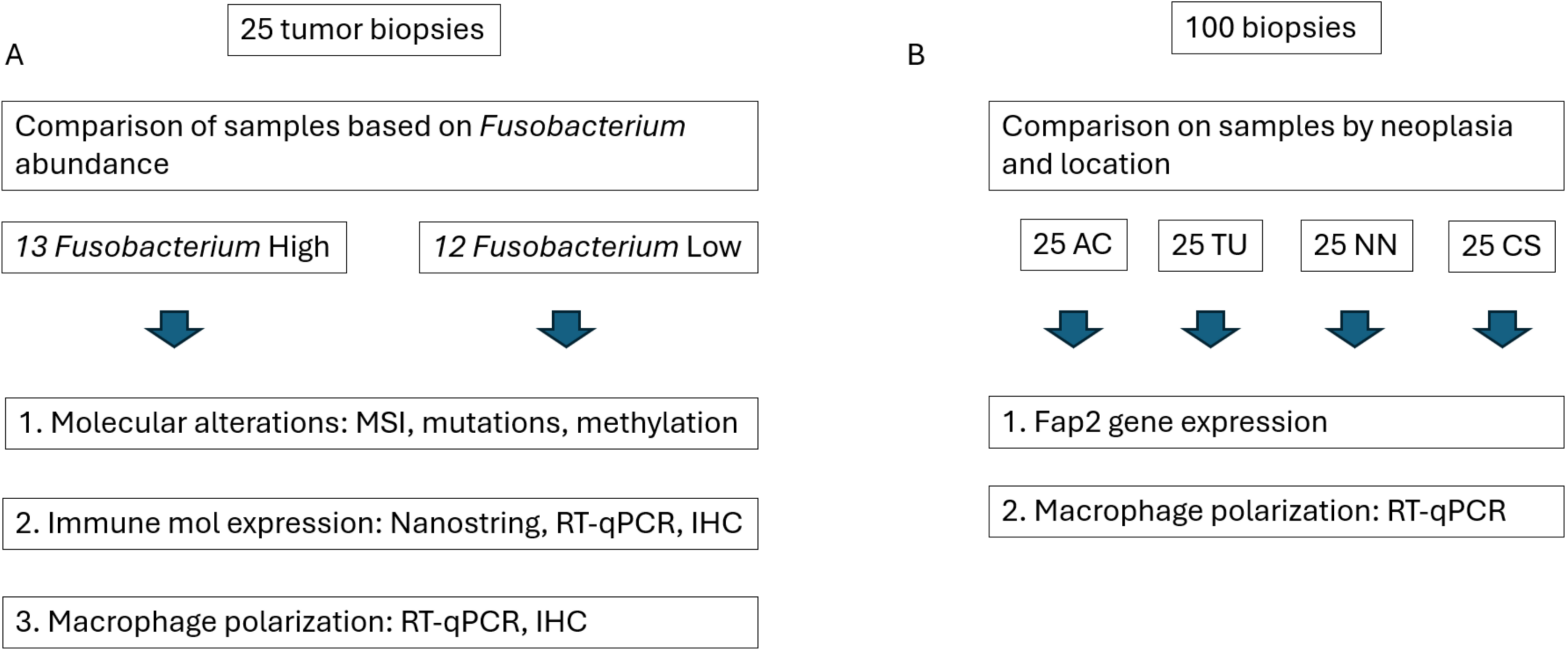
Study workflow. **(A)** Tumor samples from 25 patients were stratified into two groups based on *Fusobacterium* abundance and analyzed for differences in molecular alterations, immune molecule expression (at both gene and protein levels), and macrophage polarization. **(B)** Additional biopsies from different colonic locations—ascending colon (AC), tumor tissue (TU), non-neoplastic tissue (NN), and colon sigmoideum (CS)—were compared to assess *Fusobacterium fap2* gene expression and the expression of human genes associated with macrophage polarization.

*Fusobacterium* contents in tumor samples from 25 CRC patients were previously determined by Senthakumaran et al. (8) and Tunsjø et al. (28). *Fusobacterium* load was quantified using real-time PCR targeting the *Fusobacterium nusG* gene and normalized to the human beta-globin gene as described by Flanagan et al. (28) and Tunsjø et al. (31). Based on these data, tumor samples were divided into two groups: *Fusobacterium* high and *Fusobacterium* low, forming the basis for comparisons in the present study. The two groups were compared with respect to molecular changes, immune gene and protein expression, and macrophage polarization (**Figure 1**). Additionally, the relative abundance of *Fusobacterium* compared to the total abundance of bacteria in tumor samples and species-level identification of *Fusobacterium* were previously assessed using 16S rRNA and *zinc protease* amplicon sequencing, respectively (8). These data were used for correlation analyses in the present study.

### 2.3 Molecular characterization of CRC tumors

DNA from 25 tumor biopsies from cancer patients was analyzed for somatic mutations in the following genes: Kirsten rat sarcoma viral oncogene homolog (*KRAS*), v-raf murine sarcoma viral oncogene homolog B1 (*BRAF*), and phosphatidylinositol-4,5-bisphosphate 3-kinase catalytic subunit alpha (*PIK3CA*). Microsatellite status and methylation status of the promoter regions of MutL protein homolog 1 (*MLH1*) and O-6-Methylguanine-DNA Methyltransferase (*MGMT*) were also determined. Analysis of *KRAS* and *BRAF* mutations was performed on Rotor Gene Q v.2.3.1 (Qiagen) using the *KRAS* Mutation Analysis Kit for Real-Time PCR (EntroGen, Woodland Hills, USA) and the therascreen^®^ BRAF RGQ PCR Kit (Qiagen) according to the manufacturer’s instructions. For *PIK3CA*, the DxS PI3K Mutation Test Kit (Qiagen) was used. *BRAF* and *PIK3CA* status were determined by calculating ΔCt between amplification curves in the exons of interest and a neighboring exon without known variations. MSI status was determined by fragment analysis of selected repetitive regions of the genome using capillary gel electrophoresis with the MSI Analysis System, Version 1.2 (Promega, Madison, USA) on the 3130xl Genetic Analyzer (Applied Biosystems, Massachusetts, USA). Methylation analysis of the *MLH1* and *MGMT* promoters was done using MS-HRM (32). Primer sequences can be found in **Supplementary Table 1C**. DNA samples were bisulfite-treated and purified using the EpiTech Fast Bisulfite kit (Qiagen) per manufacturer instructions. PCR and MS-HRM were done with RotorGene Q (Qiagen). Qiagen HotStar Plus Taq polymerase was used for PCR. All PCR reaction mixes included 1 X PCR buffer with 4 mM Mg^2+,^ 200 µM dNTPs, 5 µM Syto9 (Thermo Fisher Scientific), and 200 µM of each primer. The PCR cycling conditions were 1 hold at 95°C for 5 min and 50 cycles at 95°C, 61°C, and 72°C for 5, 5, and 10 sec, respectively. HRM increased 0.1°C per 2 sec from 70 to 95°C. A standard curve with increasing allele methylation standards (0, 1, 10, 50, and 100%) was used to determine patient samples’ methylation degrees. The standards were made by mixing CpGenome Universal Methylated DNA (Zymo Research) with Universal Unmethylated DNA at the specified methylation range.

### 2.4 nCounter differential immune gene expression related to *Fusobacterium* abundance

We have previously established human immune gene expression profiles in the same 25 tumor samples employing NanoString Technologies’ nCounter Human Immunology v2 panel, featuring 579 genes related to immune response and inflammation, along with 15 housekeeping genes. Every step was conducted according to the manufacturer’s guidelines, as previously described (22). For the current study, nCounter raw data from tumor samples were re-analyzed and divided into two groups according to *Fusobacterium* load. The nCounter analysis of data was conducted using ROSALIND^®^ (San Diego, CA). Normalization, fold changes, and p-values were determined based on the criteria set forth by NanoString. ROSALIND^®^ adheres to the nCounter^®^ Advanced Analysis protocol, which involves dividing counts within a lane by the geometric mean of the normalizer probes from that same lane. Housekeeping probes intended for normalization are chosen using the geNorm algorithm as applied in the NormqPCR R library. Fold change and p-values were determined using the rapid approach outlined in the nCounter^®^ Advanced Analysis 2.0 User Manual. The Benjamini-Hochberg method was utilized for adjusting P-values to estimate false discovery rates (FDR).

Additionally, normalized counts from nCounter were plotted against the relative abundance of *Fusobacterium* reads established from 16S rRNA NGS data. GraphPad Prism 10 software (GraphPad Software, Inc., La Jolla, USA) was used for Spearman correlation analysis to assess the correlation between the expression of immune-related genes and *Fusobacterium* relative abundance.

### 2.5 Analysis of immune gene expression by real-time RT-PCR

Previous and new RT-qPCR data were utilized to validate the associations between *Fusobacterium* abundance and the expression of selected immune genes: secreted phosphoprotein 1 (*SPP1*), indoleamine 2,3-dioxygenase 1 (*IDO1*), *CXCL8*, and *IL6*. To investigate M2 macrophage polarization, RT-qPCR experiments were conducted utilizing assays that targeted the genes *cyclin D1*, cellular myelocytomatosis oncogene (*c-myc*), *ARG1*, *IL10*, interferon-gamma (*IFN-γ*), C-type lectin receptor (*CD209*), mannose receptor C-type 1 (*MRC1*/*CD206*), and inducible nitric oxide synthase (*iNOS*) across all 25 CRC patient samples collected from four locations: AC, TU, NN, and CS. We used pre-constructed TaqMan assays from Applied Biosystems and the SuperScript™ III One-Step RT-PCR System with Platinum™ Taq DNA Polymerase from Invitrogen (Thermo Fisher Scientific), with each reaction containing 10 ng of total RNA, as previously described (22). Glyceraldehyde 3-phosphate dehydrogenase (*GAPDH*) and polymerase (RNA) II (DNA-directed) polypeptide A (*POLR2A*) were used as reference genes based on previous evaluations (22). Transcription profiles were analyzed through the 2^-ΔCt^ method (33). The Shapiro-Wilk test was performed to evaluate the normality of the data. The statistical analysis involved the Kruskal-Wallis test to evaluate overall differences among groups and the Mann-Whitney U test, with P values adjusted using Bonferroni correction, to assess specific pairwise comparisons. A list of the TaqMan assays used can be found in **Supplementary Table 1A**.

### 2.6 *Fusobacterium fap2* real-time-PCR

Real-time PCR and RT-PCR were performed to evaluate the presence and gene expression of *fap2* in biopsy samples taken from four sites: AC, TU, NN, and CS from 25 CRC patients. Due to the sequence diversity of the *fap2* gene in different *Fusobacterium* species, two different sets of primers and probes were developed; one set was designed to detect *fap2* from *F. nucleatum*, *F. vincentii*, and *F. animalis* (Fap2all), while the other set was designed to specifically detect *F. animalis fap2* (Fap2an). Degenerate bases in selected positions ensured adaptability to sequence variations (**Supplementary Table 2**). All primers and probes are shown in **Supplementary Table 2A**. For detection of *Fusobacterium* RNA, the real-time RT-PCR reaction mix contained 500 nM of each primer and 250 nM probe and reagents from the SuperScript™ Platinum™ One-Step RT-PCR kit (Invitrogen, Thermo Fisher Scientific). Amplification was performed using the QuantStudio 5 qPCR instruments (ThermoFisher Scientific), and the thermal conditions were set to 20 minutes of reverse transcription at 50°C, 2 minutes of activation of Taq DNA polymerase at 94°C, followed by 40 PCR cycles at 94°C for 15 seconds and 60°C for 30 seconds. Undiluted RNA was used for testing. DNA contamination was evaluated by testing the RNA without the reverse transcription step. For the detection of *Fusobacterium* genomic DNA, PCR was performed with Brilliant III Ultra-Fast QPCR Master Mix (Agilent, Santa Clara, CA, USA) and the same temperature settings, except for the reverse transcription step. Undiluted DNA was used as a template. Specificities of the *fap2* PCR assays were evaluated using different strains of *Fusobacterium* and *Leptotrichia* and confirmed that the Fap2an PCR was specific for the *fap2* gene of *F. animalis*, while the Fap2all PCR detected *fap2* of *F. nucleatum*, *F. animalis*, and *F. vincentii* (**Supplementary Table 2B**). For gene expression analysis, the *Fusobacterium nusG* gene was used for normalization using the 2^-ΔCt^ method (8). Additionally, the total abundance of *fap2* and *nusG* RNA in each sample was extrapolated from a standard curve established with a 10-fold dilution series of RNA from *F. animalis* CCUG 32879T. The *fap2* gene expression and *fap2* and *nusG* RNA levels were correlated to different immune gene expression data using Spearman’s rank for nonparametric data.

### 2.7 Immunohistochemistry

Formalin-fixed paraffin-embedded (FFPE) tumor biopsies from 25 CRC patients were cut into slices at a thickness of 3-4 μm, deparaffinized, and rehydrated. Immunohistochemistry staining for CXCL8, c-Myc, CD163 (M2 macrophages), and CD68 (macrophages and myelomonocytic cells) was conducted at the pathology department at Akershus University Hospital. Hydrogenation, deparaffinization, and epitope unmasking were performed using a fully automated instrument, the Dako Omnis PT Link (Agilent, CA, USA). The tissue sections were stained automatically using AutostainerLink 48 from Dako (Agilent). The whole process was executed in accordance with the manufacturer’s procedure. **Supplementary Table 1B** provides a comprehensive summary of antibody clones.

To evaluate the immunohistochemical results, the assessment of the staining was based on the method proposed by Shao et al. (23), as follows: Staining intensity was graded as 0 (no staining), 1 (weak staining), 2 (moderate staining), and 3 (intense staining). The percentage of positively stained cells was scored as 0 (0%), 1 (1%–49%), 2 (50%–89%), and 3 (90%–100%). To allow numerical data that could be used for statistical comparison, the immunohistochemical score was obtained by multiplying the intensity and the percentage scores of the antibodies, which ranged from 0 to 9 (23). The whole biopsy was evaluated, and this was done by two independent pathologists at Akershus University Hospital. For statistical comparison between groups of *Fusobacterium* High and Low, the Mann-Whitney U test was conducted.

## 3 Results

### 3.1 Molecular characteristics of the tumors and their association with *Fusobacterium* abundance

We aimed to investigate the potential relationship between *Fusobacterium* and somatic mutations in CRC-associated genes in a Norwegian cohort of 25 CRC tumors. Based on the *Fusobacterium nusG* quantitation, 13 samples were categorized as *Fusobacterium* high, and 12 samples were categorized as *Fusobacterium* low (**Table 2**). Among the thirteen *Fusobacterium* high samples, nine contained *F. animalis* (**Table 2**). Three other *Fusobacterium* species, *F. nucleatum*, *F. vincentii*, and *F. pseudoperiodonticum*, were also identified in this group, as well as unidentified *Fusobacterium* species. In the *Fusobacterium* low group, *Fusobacterium* was detected at low levels in eight samples. Specifically, *F. animalis* was found in two samples, while one sample harbored *F. gonidiaformans* and another *F. necrophorum.* Additionally, four samples contained low levels of unidentified *Fusobacterium* sp. (**Table 2**).

**Table 2:** *Fusobacterium* and molecular characteristics of the tumor samples.

Sample	*Fusobacterium*		Mutation status			MSS/ MSI*	Gene promoter methylation (%)	
	Species (8)	Load (28)	KRAS	BRAF	PIK3CA		MGMT	MLH1
C01	*Fusobacterium* sp.	High	Neg	Neg	Neg	MSS	25	0
C03	*F. animalis*		Neg	V600E	Neg	MSI High	25	50
C04	*F. animalis*		Neg	Neg	Neg	MSS	0	0
C05	*Fusobacterium* sp.		G12S	Neg	Neg	MSS	10-25	0
C06	*Fusobacterium* sp. *F. animalis* *F. vincentii*		Neg	V600E	Neg	MSI High	10-25	50
C08	*F. animalis*		Neg	Neg	Neg	MSI High	0	25
C09	*F. animalis*		Neg	V600E	Neg	MSS	0	0
C11	*Fusobacterium* sp. *F. animalis*		Neg	V600E	Neg	MSI High	25-50	50
C12	*F. pseudoperiodonticum*		Neg	Neg	Neg	MSS	0	0
C13	*F. animalis*		Neg	Neg	Neg	MSS	0	0
C15	*F. animalis*		Neg	Neg	Neg	MSS	0	0
C19	*F. animalis*		Neg	Neg	H1047R	MSI High	0	0
C24	*F. nucleatum*		Neg	Neg	Neg	MSS	50-100	0
C02	*F. necrophorum*	Low	Neg	Neg	Neg	MSS	0	0
C07	*Fusobacterium* sp.		G12D	Neg	Neg	MSS	≈ 5	0
C10	Not detected		G12S	Neg	Neg	MSS	0	0
C14	*F. animalis*		Neg	Neg	Neg	MSS	0	0
C16	Not detected		G12V	Neg	Neg	MSS	0	0
C17	Not detected		Neg	Neg	Neg	MSS	0	0
C18	*Fusobacterium* sp.		Neg	Neg	Neg	MSS	0	0
C20	Not detected		G12S	Neg	H1047R	MSS	0	0
C21	*Fusobacterium* sp.		Neg	Neg	Neg	MSS	5	0
C22	*F. animalis*		A146X	Neg	Neg	MSS	10-25	0
C23	*F. gonidiaformans*		Neg	Neg	E542K	MSS	0	0
C25	*Fusobacterium* sp.		Neg	Neg	Neg	MSS	25	0

*MSS, Microsatellite stable; MSI, Microsatellite instability. There is a significant association between *Fusobacterium* load and MSI status based on the chi-square test (p = 0.016) and Fisher’s exact test (p = 0.039).

The samples were analyzed for *KRAS*, *BRAF*, and *PIK3CA* mutations as well as MSI status and methylation of the *MLH1* and *MGMT* genes. All samples exhibiting MSI high status were located within the *Fusobacterium* high group (**Table 2**), and a significant association was observed, as demonstrated by the chi-square test (p = 0.016) and Fisher’s exact test (p = 0.039). Just a single sample with MSI-H lacked *MLH1* or *MGMT* promoter methylation. Next-generation sequencing (NGS) with the Oncomine Comprehensive Assay V2 revealed a truncating mutation in the *PMS2* gene (*PMS1* homolog 2), a major component of the mismatch repair (MMR) mechanism. The mutation had a variant frequency of 25%, suggesting a somatic mutation rather than a germline mutation (data not shown). All four MSI-H samples containing *BRAF*–*V600E* mutations were in the *Fusobacterium*-high load group, but there was no statistically significant association. Six samples were found to contain *KRAS* mutations, including G12S, G12D, and G12V. Except for a single sample, every sample exhibiting *KRAS* mutations was found in the *Fusobacterium* Low group. The one sample with a *KRAS* mutation identified within the *Fusobacterium*-high group was *F. nucleatum* positive. Three samples displayed *PIK3CA* mutations, but no association to *Fusobacterium* load was identified (**Table 2**).

### 3.2 Differentially expressed genes and correlation with *Fusobacterium* abundance

**Figure 2:**
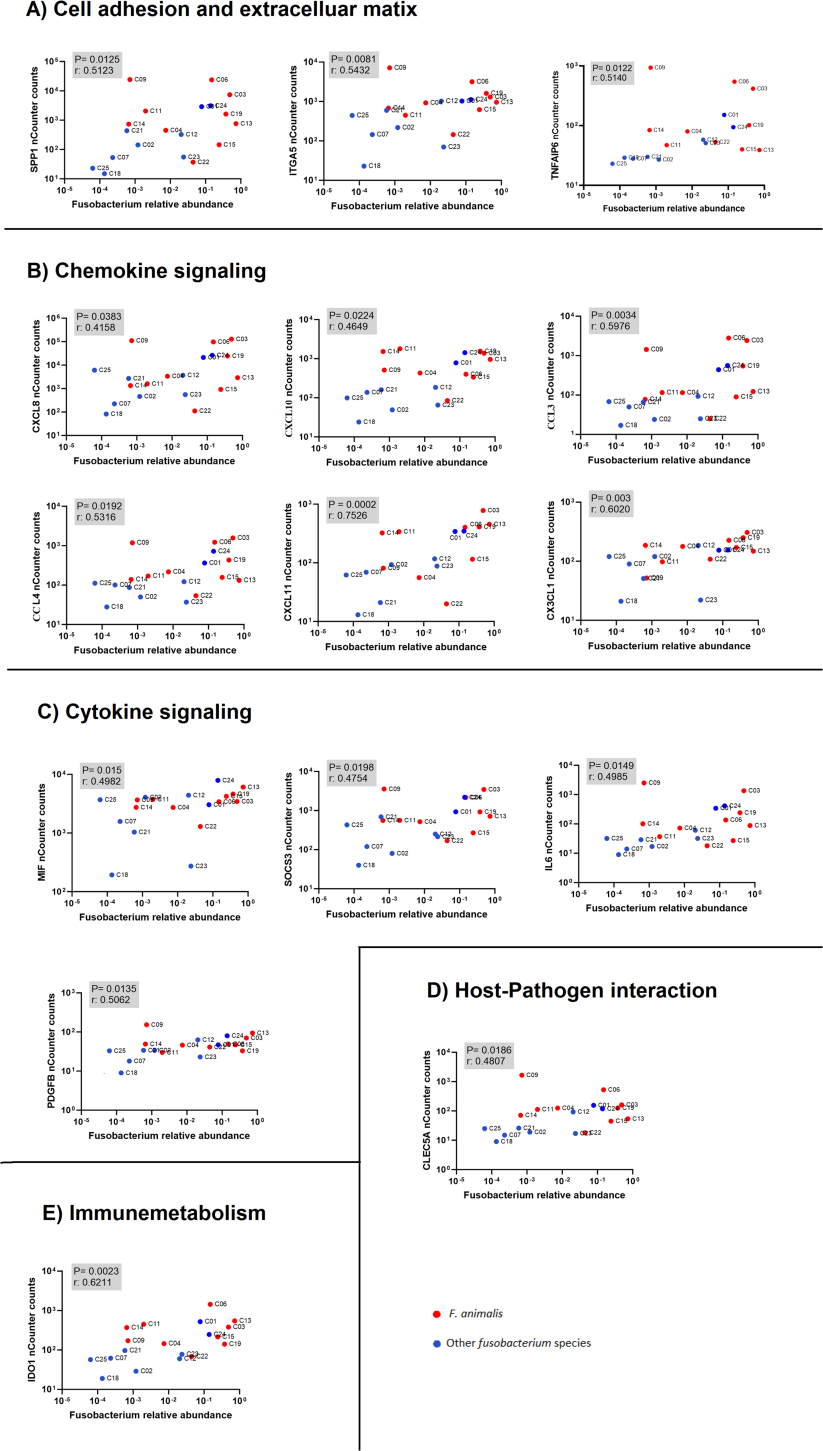
Correlation analysis between immune gene expression (nCounter counts from NanoString) and *Fusobacterium* relative abundance (NGS-based quantification). The plots show associations between immune-related genes and *Fusobacterium* species, with red dots representing *F. animalis* and blue dots representing other *Fusobacterium* species. The human genes are categorized into functional groups: **(A)** cell adhesion and extracellular matrix, **(B)** chemokine signaling, **(C)** cytokine signaling, **(D)** host-pathogen interaction, and **(E)** immunometabolism. Each plot displays the p-value and correlation coefficient (r) to indicate the statistical significance and strength of the correlation.

Data from the nCounter Human Immunology V2 panel were analyzed to identify differentially expressed genes between high- versus low-*Fusobacterium*-load groups using a Mann–Whitney U test. Next, we correlated sample-wise mRNA counts from the nCounter assay with *Fusobacterium* relative abundance determined by Illumina 16S rRNA sequencing, as reported in an earlier publication using the same samples (8). By using two methodologically distinct approaches to estimate *Fusobacterium* load in CRC tumors, we identified 34 immune genes that were statistically significant with both approaches. However, we have previously shown in our earlier publication, Omran et al., 2024, that nine of these genes display equal expression levels in adjacent non-cancerous tissue (22). The final analysis identified 25 immune genes that were highly expressed in tumor samples and displayed statistically significant association to *Fusobacterium* load with two distinct approaches (**Table 3**, **Figure 2**). Complete gene lists and associated data are provided in **Supplementary Tables 3** and **4** for the *Fusobacterium* high/low analysis and correlation analysis, respectively. Among these genes, *CXCL8* was notable for its exceptionally high average nCounter counts in the *Fusobacterium* high group, showing a 27-fold change compared to the *Fusobacterium* low group. Other genes like *SPP1*, chemokine (C-C motif) ligand 3 (*CCL3)*, *IL6*, and *IDO1* also displayed a high average count in the *Fusobacterium* high group as well as a relatively high fold change between the groups (**Table 3**). The correlation between *Fusobacterium* relative abundance and top 15 immune gene counts is shown in scatter plots (**Figure 2**). Notably, the samples exhibiting elevated immune gene expression contained not only *F. animalis* but also various other *Fusobacterium* species (**Figure 2**).

**Table 3:** Comparison analysis of immune genes associated with *Fusobacterium* in CRC.

Cellular function	Gene name	Correlation with *Fusobacterium* relative abundance*		DEG *Fusobacterium* high/low**			
		r	P-value	Fold change	p-Adj	Mean counts high	Mean counts low
Antigen presentation	TAP1	0.42	<0.05	2.2	<0.05	803	266
Cell adhesion and extracellular matrix	SPP1	0.51	<0.05	15.8	<0.05	5547	219
	ITGA5	0.54	<0.01	5.0	<0.01	1657	321
	ICAM1	0.39	<0.05	5.3	<0.01	1063	206
	TNFAIP6	0.51	<0.05	7.2	<0.01	253	40
Chemokine signaling	CXCL8 (IL8)	0.42	<0.05	26.7	<0.01	39121	1510
	CXCL10	0.46	<0.05	5.4	<0.01	1224	247
	CCL3	0.60	<0.01	17.4	<0.01	823	57
	CCL4	0.53	<0.01	6.4	<0.01	634	94
	CXCL11	0.75	<0.001	8.2	<0.01	585	85
	CX3CL1	0.60	<0.01	2.1	<0.05	165	95
Cytokine signaling	MIF	0.50	<0.05	1.7	<0.05	3982	1898
	SOCS3	0.48	<0.05	4.2	<0.05	1509	302
	IL6	0.50	<0.05	14.7	<0.05	577	32
	IL1RAP	0.46	<0.05	2.3	<0.05	202	84
	TNF	0.53	<0.05	2.7	<0.05	159	60
	PDGFB	0.51	<0.05	2.6	<0.05	64	31
Host-pathogen interaction	CLEC5A	0.48	<0.05	22.4	<0.01	278	28
	GBP5	0.42	<0.05	4.8	<0.01	225	54
	NLRP3	0.51	<0.05	5.6	<0.05	131	35
Immunometabolism	IDO1	0.62	<0.01	11.2	<0.01	916	98
Lymphocyte activation	CD80	0.45	<0.05	2.3	<0.05	104	44
	LILRB3	0.43	<0.05	4.0	<0.05	102	33
	LILRA3	0.56	<0.01	5.6	<0.05	101	27
Transcriptional regulation	BCL6	0.40	<0.05	2.6	<0.05	240	85

*Relative abundance established with 16S rRNA amplicon sequencing. *Fusobacterium* reads were normalized to the total number of bacterial reads (8).

**Samples were divided into groups of *Fusobacterium* high and low based on qPCR quantitation with the *Fusobacterium-*specific *nusG* gene (28).

**Figure 3:**
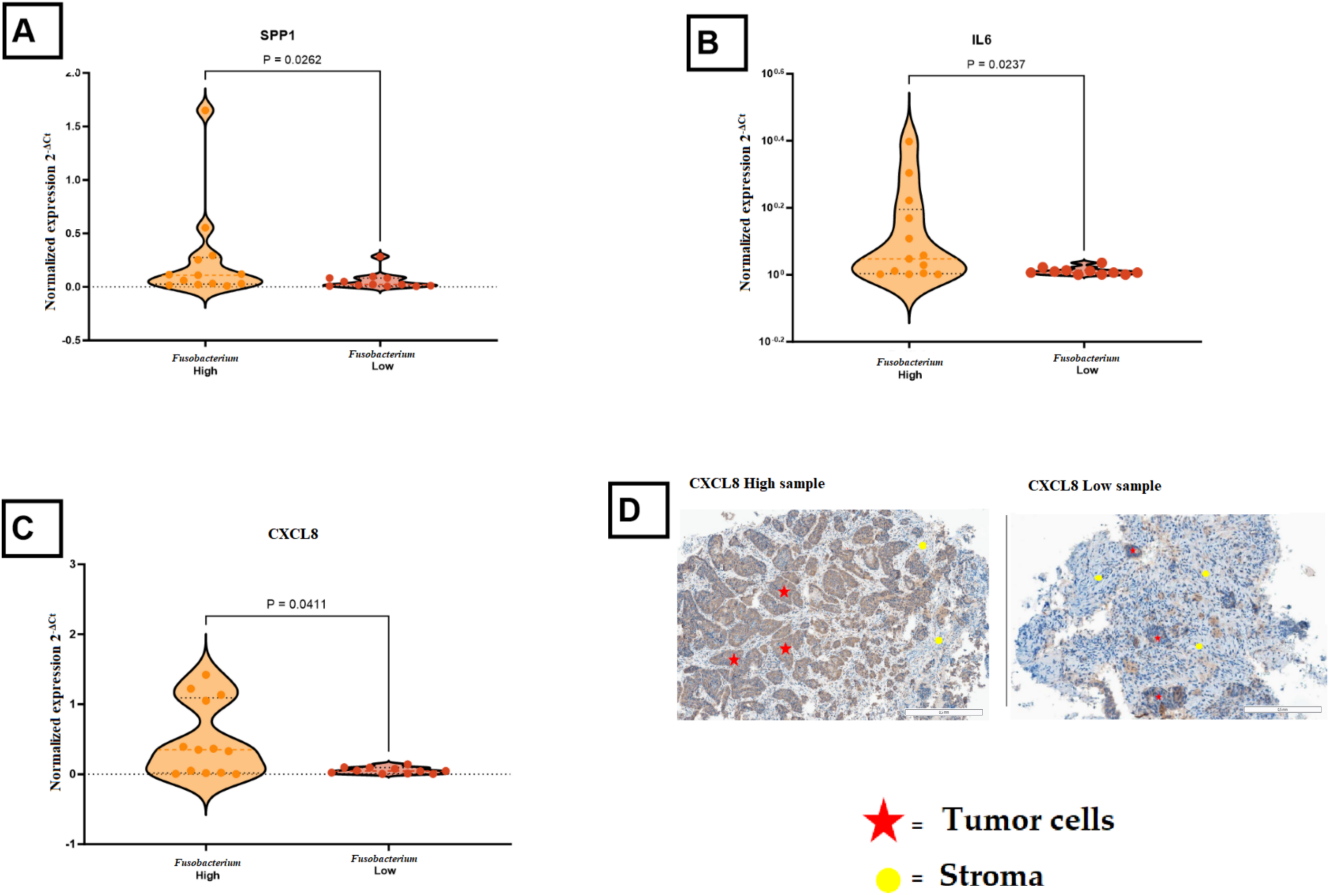
Comparison of immune gene expression in *Fusobacterium*-high and *Fusobacterium*-low colorectal cancer samples. **(A)** *SPP1* expression was significantly higher in *Fusobacterium*-high samples compared to *Fusobacterium*-low samples (P = 0.0262). **(B)** *IL6* expression was elevated in *Fusobacterium* high samples (P = 0.0237). **(C)** *CXCL8* showed increased expression in *Fusobacterium* high samples (P = 0.0411). **(D)** Representative immunohistochemical (IHC) images of high and low *CXCL8* expression in tumor tissues, illustrating that *CXCL8* is mainly produced by tumor cells. Red stars indicate tumor cells, and yellow circles mark stromal regions.

To confirm DEG and correlation results, RT-qPCR analysis was performed for selected immune genes that displayed high expression levels and different biological functions. The expression levels of immune genes (*IDO1*, *SPP1*) and pro-inflammatory signaling genes (*IL6*, *CXCL8*) were confirmed as markedly elevated in *Fusobacterium*-high samples. Statistical analysis revealed significant differences for *SPP1* (p = 0.0028), *IL6* (p = 0.0237), and *CXCL8* (p = 0.0411) (**Figures 3A–C**). *IDO1* exhibited elevated levels in the *Fusobacterium-*high group, although this was not statistically significant (p-value 0.06) (data not shown). Analysis of CXCL8 expression through immunohistochemistry showed that CXCL8 was primarily found in tumor cells, showing a trend of increased but not statistically significant CXCL8 expression in *Fusobacterium*-high tumors (**Figure 3D**).

### 3.3 *Fusobacterium* adhesin *fap2* in colorectal tumors

**Figure 4:**
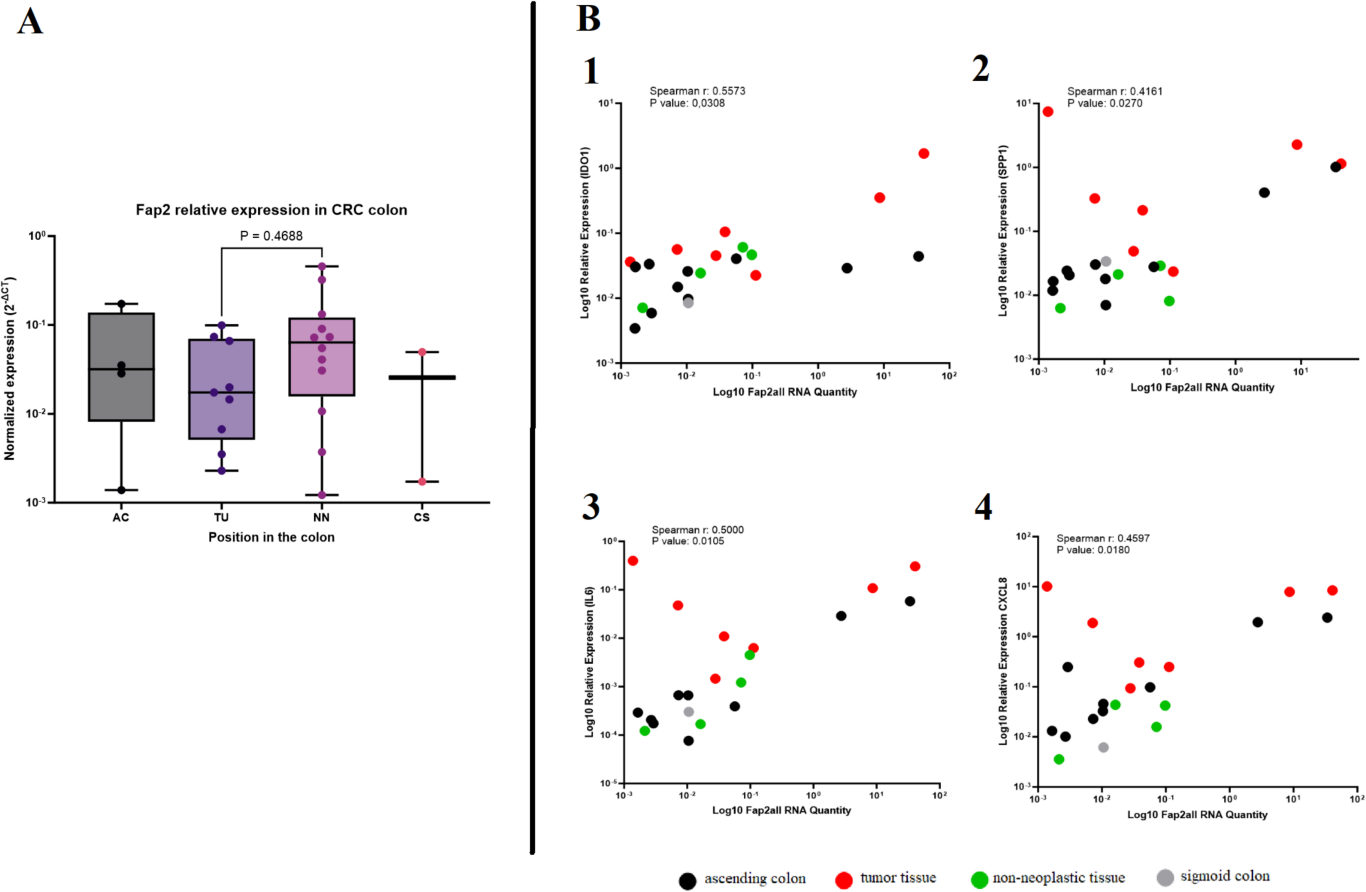
The *fap2* gene and its correlation with immune gene expression in colorectal cancer (CRC) tissues. **(A)** The box plot shows normalized *fap2* gene expression in different regions of the colon, including the ascending colon (AC), tumor tissue (TU), non-neoplastic tissue (NN), and sigmoid colon (CS). No significant difference was observed between tumor and non-neoplastic tissues (P = 0.4688). **(B)** Correlation analysis between *fap2* RNA levels and immune gene expression. Expression of the four investigated genes was positively correlated with *fap2* RNA quantity: IDO1 (Spearman r = 0.5573, P = 0.0308), *SPP1* (Spearman r = 0.4161, P = 0.0270), *IL6* (Spearman r = 0.5000, P = 0.0105), and *CXCL8* (Spearman r = 0.4597, P = 0.0180). Colored dots indicate different tissue types: tumor tissue (red), ascending colon (black), non-neoplastic tissue (green), and sigmoid colon (gray).

To gain more insight into the *Fusobacterium* virulence factor Fap2 in the tumor microenvironment, PCR and RT-PCR were performed to detect *fap2* presence (DNA) and expression (RNA), respectively. *fap2* DNA was detected in biopsy samples from ten out of twelve patients with *F. animalis* or *F. nucleatum*, illustrating that *fap2* was present in the majority of samples containing *Fusobacterium* species that were detectable by either of the two *fap2* PCR assays. *fap2* RNA was detected in tumor and adjacent non-neoplastic tissue from the same patients, although with higher Ct-values, confirming active *fap2* transcription. *fap2* RNA was only detected in three of six *fap2* DNA-positive samples from AC and in one of six *fap2* DNA-positive samples from CS, possibly due to RNA levels below the limit of detection (> 40 Ct) or reduced gene expression. These high Ct values (> 40) suggest RNA levels were close to the detection limit, and therefore the possibility of false-negative results cannot be excluded. The gene expression of *fap2* was compared across different regions of the colon in cancer patients using the 2^-ΔCt^ method and *nusG* as a reference gene. Only minor and non-significant differences in gene expression between tumor tissues and adjacent non-neoplastic tissues were observed, suggesting constitutive expression of *fap2*. The sample sizes were insufficient for a comparative analysis of expression levels in AC and CS (**Figure 4A**). A correlation analysis was then performed between *fap2* gene expression (2^-ΔCt^) and gene expression of the chosen immune genes *IDO1*, *SPP1*, *CXCL8*, and *IL6* using RT-PCR data. The correlations were assessed in four distinct areas of the colon, with the majority of samples from tumor tissue as well as neighboring non-neoplastic regions. The 2^-ΔCt^ method with *nusG* as a reference gene showed no correlation between the expression of *fap2* and the different immune genes, illustrating that *fap2* expression was not induced above normal levels in the inflamed tumor environment. However, a positive correlation was found between total abundance of *fap2* RNA and expression of *IDO1* (r = 0.5573, p = 0.0308), *SPP1* (r = 0.4161, p = 0.0270), *IL6* (r = 0.5000, p = 0.0105), and *CXCL8* (r = 0.4597, p = 0.0189) (**Figure 4B**). A positive correlation was also found between *nusG* RNA levels and the same immune genes, illustrating that the positive correlations are a result of *Fusobacterium* RNA abundance and generally active transcription.

### 3.4 M2 macrophage infiltration in colorectal tumors and their association with *Fusobacterium* load

The immunology V2 nCounter panel used in this study includes several genes specifically expressed by different types of immune cells and provides an opportunity to identify the presence of different immune cells and immune cell differentiation in the samples studied. However, the nCounter counts of immune cell-specific markers like *CD4*, *CD8*, *CD209*, and *CD206* were relatively low across the samples in our study and would not provide the detection of specific immune cells, probably because the relative contribution of mRNA from immune cells in samples that mainly consist of tumor cells is low. As RT-PCR might be more sensitive than nCounter analyses, we therefore used RT-PCR to investigate the expression of several genes associated with M1 and M2 polarization of macrophages.

**Figure 5:**
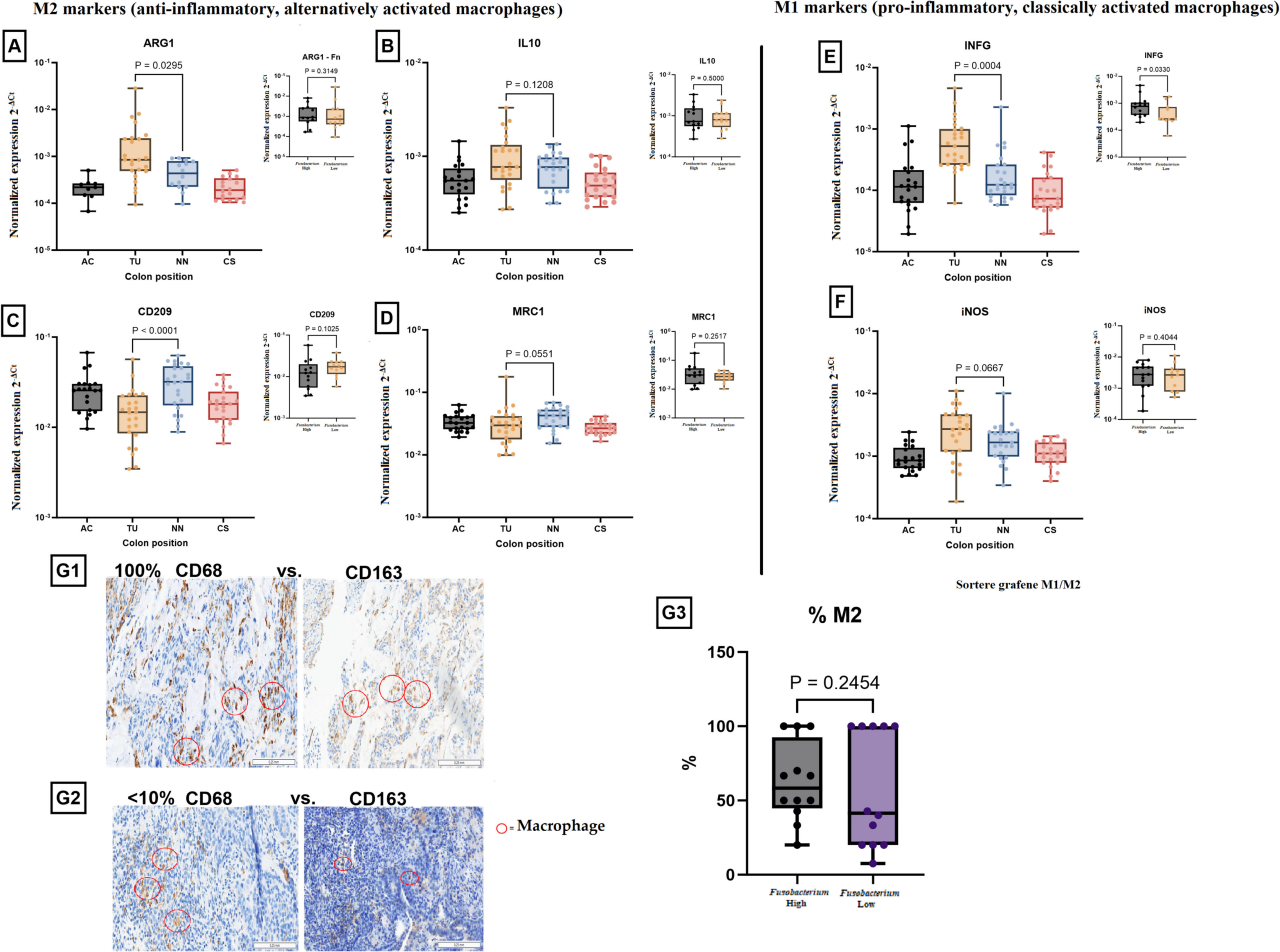
Expression of M1 and M2 macrophage markers in colorectal cancer (CRC) and their association with *Fusobacterium* load. M2 markers (anti-inflammatory, alternatively activated macrophages): **(A)** *ARG1* expression was significantly higher in tumor tissue (TU) compared to other regions (P = 0.0295). A separate analysis (inset) showed no significant difference based on *Fusobacterium* load (P = 0.3148). **(B)** *IL10* expression showed no significant difference across colon regions (P = 0.1208) or between *Fusobacterium* high and *Fusobacterium* low groups (P = 0.5002). **(C)** *CD209* expression was significantly upregulated in non-neoplastic tissue (P < 0.0001) but showed no significant association with *Fusobacterium* (P = 0.1205). **(D)** *MRC1* expression showed a trend towards higher expression in the non-neoplastic tissue (NN) (P = 0.0551), with no significant difference in *Fusobacterium* high versus *Fusobacterium* low samples (P = 0.2517). M1 markers (pro-inflammatory, classically activated macrophages): **(E)** *IFNG* expression was significantly higher in tumor tissue (P = 0.0004), but no significant difference was observed between the *Fusobacterium*-high and *Fusobacterium*-low groups (P = 0.6830). **(F)** *iNOS* expression showed a trend towards increased expression in tumor tissue (P = 0.0667), with no significant difference between *Fusobacterium* high and *Fusobacterium* low groups (P = 0.4044). Immunohistochemical (IHC) analysis of macrophage polarization: **(G1)** Example of a tumor sample with a proportion of CD163-positive macrophages compared to CD68-positive macrophages of 100%. **(G2)** Example of a tumor sample with a proportion of CD163-positive macrophages compared to CD68-positive macrophages of < 10%. **(G3)** Quantification of M2 macrophage percentage in *Fusobacterium* high versus *Fusobacterium* low samples. No significant difference was observed (P = 0.2454). Red circles highlight macrophages in IHC images.

RT-PCR analysis of key M1 and M2 macrophage markers was conducted using samples from the ascending colon (AC), tumor tissue (TU), adjacent normal tissue (NN), and sigmoid colon (CS). Among the analyzed markers, *IL10, MRC1* (*CD206*), and *iNOS* did not show statistically significant differences between the tissue types or between *Fusobacterium* high and low tumors. However, significant differences were observed for several other markers. *ARG1* expression (an M2 macrophage marker) was markedly elevated in TU tissues relative to NN tissue (P = 0.0295, **Figure 5A**). Nonetheless, *ARG1* expression exhibited no significant variation across tissues with elevated *Fusobacterium* load. Interferon gamma (*INFG*) expression was markedly elevated in TU tissues relative to NN and CS (P = 0.0004, **Figure 5C**) and was increased in tissues with high *Fusobacterium* load compared to those with low load (P = 0.0330, **Figure 5C**). *CD209* expression (an M2 macrophage marker) was markedly reduced in TU tissues relative to NN (P < 0.0001, **Figure 5D**). Nonetheless, no substantial difference was detected between high and low *Fusobacterium* load groups. To evaluate M2-macrophage polarization, immunohistochemical analysis was performed for CD163 (M2 marker) and CD68 (pan-macrophage marker). The quantification of M2 macrophages (%M2) indicated no significant differences between tissues with elevated and reduced *Fusobacterium* loads (P = 0.2454, **Figures 5G1–G3**).

### 3.5 Expression of the genes encoding *cyclin D1* and *c-myc*

**Figure 6:**
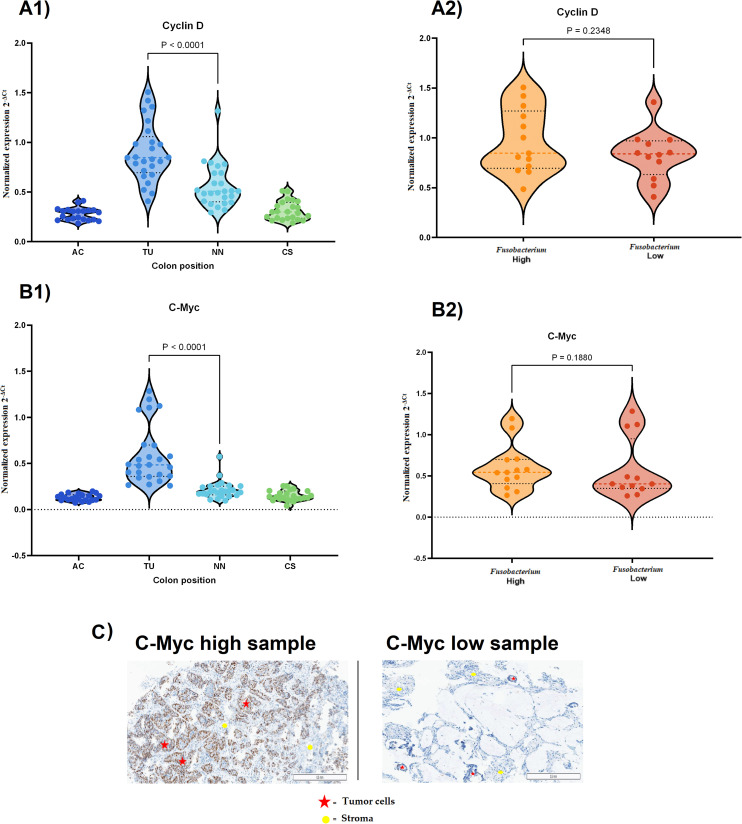
Expression of *cyclin D1* and *c-myc* in CRC tissues and their association with *Fusobacterium* load. **(A1)** *cyclin D1* expression across different colon regions in CRC samples. Expression was significantly higher in tumor tissue (TU) compared to non-neoplastic (NN) tissue and other colon regions (P < 0.0001). **(A2)** *cyclin D1* expression in *Fusobacterium*-high and *Fusobacterium*-low samples. No significant difference was observed between the two groups (P = 0.2348). **(B1)** *c-myc* expression across different colon regions in CRC samples. *c-myc* was significantly upregulated in tumor tissue (P < 0.0001). **(B2)** *c-myc* expression in *Fusobacterium* high and *Fusobacterium* low samples. No significant difference was observed (P = 0.1880). **(C)** Immunohistochemical (IHC) images of c-Myc expression in CRC tissues. The left panel shows a sample with high c-Myc expression, while the right panel shows a sample with low expression in tumor cells. Red stars indicate tumor cells, and yellow circles mark stromal regions.

To study a possible association between *Fusobacterium* and cancer cell proliferation, we analyzed the expression levels of *cyclin D1* and *c-myc* in tumor tissues relative to other tissue types, including tissue from AC, TU, NN, and CS. The results showed that these genes were significantly upregulated in TU tissues compared to NN tissues (P < 0.0001, **Figures 6A1, B1**). However, the comparison of *cyclin D1* expression between the high and low *Fusobacterium* load groups showed no significant difference (P = 0.2348, **Figure 6A2**). Similarly, no significant difference in *c-myc* expression was observed between the high and low *Fusobacterium* load groups (P = 0.1880, **Figure 6B2**). IHC examination of c-Myc protein levels in tumor cells revealed no significant difference between groups with high and low *Fusobacterium* load (P = 0.3184, **Figures 6C1, C2**).

## 4 Discussion

This study aimed to investigate *Fusobacterium*-associated molecular and immunological changes in colorectal tumor biopsies from Norwegian patients, building on our earlier findings of a significantly elevated prevalence of *Fusobacterium* within this cohort (8, 28). In this study we examined the relationship between *Fusobacterium*, *fap2*, and immune gene expression in CRC tumors. Additionally, we investigated the association of *Fusobacterium* with oncogenic mutations, microsatellite instability, and macrophage polarization.

Our study demonstrated a significant association between *Fusobacterium* and microsatellite instability-high (MSI-H) tumors. Except for one, all samples harbouring *BRAF* V600E mutations are in addition MSI-H status (**Table 2**). These findings are consistent with the previous research linking *Fusobacterium* with microsatellite instability (18, 20, 34). This association may have significant implications for CRC prognosis, as MSI-high tumors are associated with specific therapeutic responses, particularly to immune checkpoint inhibitors (35). Patients with *BRAF*-mutated CRC have a poor prognosis because their tumors are aggressive and resistant to current therapies (36). MSI-H *BRAF*-mutant tumors react better to immune checkpoint inhibitors such as anti-PD-1 therapy, which improves survival in this group (37). However, emerging evidence suggests that *Fusobacterium* infection in MSI-H cancers may impair immunotherapy. *F. nucleatum* has been shown to suppress T cell infiltration and produce an immunosuppressive tumor microenvironment, perhaps aiding immune evasion (38). Although causality remains unproven, our findings support the hypothesis that *Fusobacterium* influences CRC progression via oncogenic pathways (12, 13, 15, 18, 27).

The association analyses between *Fusobacterium* and immune gene expression identified 25 genes present in the nCounter Immunology V2 panel that were associated with high levels of *Fusobacterium* (**Table 2**). Of the 25 genes, *CXCL8* (*IL8*) stood out with the highest fold change and an exceptionally high count in the *Fusobacterium* high group, suggesting that the presence of *Fusobacterium* in tumor tissue may stimulate CXCL8 production. Subsequent immunohistochemical analyses demonstrated that CXCL8 was mainly produced by the tumor cells. This is in line with *in vitro* studies performed by Casasanta et al., who showed that a human CRC tumor cell line produces CXCL8 when incubated with a laboratory strain of *F. nucleatum* (15). Others have shown in tumor cell lines and animal models that CRC tumor cells express one of the receptors for CXCL8, C-X-C motif chemokine receptor 2 (CXCR2), and that stimulation with CXCL8 induces tumor cell growth and metastasis (39, 40). In human CRC studies, an association between CXCL8 levels and prognosis has been suggested (40–44). Taken together, our study points toward the possibility that *Fusobacterium* may promote tumor growth by inducing CXCL8 production in human CRC cancer cells. Several other chemokines were associated with *Fusobacterium*, including the CXCL chemokines C-X-C motif chemokine ligand 10 (*CXCL10*) and C-X-C motif chemokine ligand 11 *(CXCL11*) and the (C-C motif) ligand (CCL) chemokines *CCL3* and *CCL4*. CXCL10 and CXCL11 may be primarily produced by myeloid cells in the tumor microenvironment (18, 27, 45–47), and several reports suggest that CXCL10 and CXCL11 have anti-tumor effects in CRC, possibly by recruiting cytotoxic lymphocytes to the tumor (48–51). Interestingly, we observed that *Fusobacterium* load was also associated with MSI-H status and elevated levels of *IFN*γ (**Table 2**, **Figure 5**), both associated with a cytotoxic anti-tumor response. On the other hand, CXCL10 and CXCL11 have been described to enhance cancer development, possibly by promoting angiogenesis and metastasis (51, 52). Like CXCL10 and CXCL11, CCL3 and CCL4 have been attributed both tumor-promoting and anti-tumor effects (53). While this study suggests an association between *Fusobacterium* and these chemokines, the causality between *Fusobacterium* and these chemokines remains to be investigated.

The expression of *SPP1* and *IL6* was associated with *Fusobacterium* load, both displaying a high fold change in the DEG analysis. Both molecules are associated with colorectal cancer progression, where *SPP1* might promote cancer cell migration and invasion (54) and where *IL6* is a pro-inflammatory cytokine that may promote cancer cell proliferation and survival (55). The survival effects of IL6 were shown to be mainly mediated by the activation of the transcription factor STAT3 in a mouse model of colitis-associated cancer (56). Interestingly, we identified high expression of the STAT3 inhibitor, suppressor of cytokine signaling 3 (SOCS3), in CRC samples with *Fusobacterium*, suggesting that STAT3 activation is inhibited in these samples (**Table 3**). However, further investigations are needed to dissect a possible connection between *Fusobacterium* load and IL6-mediated cancer progression.

Most of the genes that were elevated in the *Fusobacterium* high group in the present study also showed increased expression in tumor samples compared to non-neoplastic tissue in our previous study, regardless of *Fusobacterium* levels (22). This indicates that *Fusobacterium* may not trigger a unique immune response beyond what is already initiated in the tumors, but it could enhance the existing response. This is in line with Udayasuryan et al., who recently showed in an *in vitro* study that CXCL8 and CXCL1 are induced under hypoxic conditions independently of *Fusobacterium* infection, with hypoxia promoting *Fusobacterium* invasion and exacerbating the hypoxia-induced effects (21).

*F. animalis* has emerged as the most prevalent *Fusobacterium* species in colorectal tumors (8, 9). However, our research, along with that of others, has identified multiple *Fusobacterium* species in tumor biopsies, albeit with lower prevalence (**Table 2**). In the present study, we observed four samples in the *Fusobacterium* high group that contained other *Fusobacterium* species. As illustrated in **Figure 2**, both *F. animalis* and other *Fusobacterium* species contributed to the correlation between *Fusobacterium* relative abundance and immune gene expression. This supports that several *Fusobacterium* species may contribute to CRC, yet the predominance of *F. animalis* in tumors suggests an underlying cause that warrants further investigation. Schmidt et al. showed that *F. animalis*, followed by *F. nucleatum*, are two of the oral species with the highest oral-fecal transmission rates, particularly in colorectal cancer patients (57). The increased transmission of *F. animalis* could possibly be related to its colonization of tumors, however; it does not explain its predominance relative to *F. nucleatum*.

The importance of Fap2 is therefore an area of interest. A better overview of Fap2 in the tumor environment may be useful for the understanding of the role of different *Fusobacterium* strains associated with CRC. Sivertsen et al. showed that not all *F. animalis* genomes contain the prototypic *fap2* gene, and others have suggested that *Fusobacterium* strains without Fap2 may not bind to tumor cells (13, 58). We therefore tested all biopsy samples for the presence of *fap2* and identified *fap2* DNA in the majority of biopsy samples from cancer patients that contained *F. animalis, F. nucleatum*, or *F. vincentii*. Its presence in these samples may indicate that *Fusobacterium* strains that colonize tumor lesions express Fap2. Since the *fap2* PCR assays did not detect *fap2* from other *Fusobacterium* species, the presence of *fap2* in these samples could not be established. We also measured *fap2* RNA and used the ΔCt method to evaluate differences in *fap2* gene expression between the different colonic sites. We found no significant differences between tumor samples and adjacent non-neoplastic tissues. Our findings therefore align with studies showing that *fap2* is constitutively expressed. Cochrane et al. observed no changes in *fap2* expression during the initial phase of infection in an *in vitro* cell model (59). Similarly, Ponath et al. created global RNA maps for five clinically relevant *Fusobacterium* species and found no variation in *fap2* expression across different *in vitro* conditions, concluding that *fap2* expression was growth independent (60). Thus, while *fap2* appears essential for infection, it does not seem to function as an inducible virulence factor regulated by quorum sensing or other environmental signals. The constitutive expression may explain its ability to colonize various body regions, including extensive portions of the colon, as shown in the present study. Although *fap2* gene expression did not correlate with activated immune genes, the total abundance of *fap2* and *nusG* RNA in each sample correlated with the expression of *CXCL8*, *IL6*, *SPP1*, and *IDO1*, suggesting that the load of active *Fusobacterium* cells is related to, and possibly influences, immune gene expression. However, the constitutive nature of *fap2* expression argues against its regulation by the inflammatory microenvironment. Taken together the bacterium’s association with pro-inflammatory gene expression in colorectal cancer and supports the notion that *Fusobacterium* has a role in the stimulation of the observed immune response (26, 27).

Additionally, we investigated *Fusobacterium-*associated polarization of TAMs in colorectal cancer tumors. TAMs play an important role in tumor progression and metastasis. Macrophages are highly versatile immune cells that exhibit significant heterogeneity and plasticity, adapting to various signals within the TME (61). In CRC, macrophages can adopt different phenotypes, primarily classified as M1 and M2 macrophages, each playing distinct roles in tumor progression. M1 macrophages produce pro-inflammatory cytokines such as INF-γ and TNF-α, which can inhibit tumor growth and promote tumor immunity. On the other hand, M2 macrophages secrete anti-inflammatory cytokines such as IL-10 and TGF-β that promote tissue repair, angiogenesis, and tumor progression (24). The impact of *Fusobacterium* infection on M2 polarization of macrophages has been investigated *in vitro*, as well as in Apc ^Min^/^+^ mice and in CRC tumor biopsies (26, 27). Chen et al. used immunofluorescent labeled antibodies against M1 and M2 macrophages (anti-CD86 and anti-CD206, respectively) and showed significantly higher amounts of M2 macrophages in *F. nucleatum*-positive tumor biopsies (27). However, our results are contradictory, showing that the increased amounts of M2 macrophages are not associated with the abundance of *Fusobacterium* in the tumor biopsies (**Figures 5G1–G3**). We further examined the tumor samples for expression of known M1 and M2 macrophage markers using quantitative RT-PCR assays, and the results showed no significant differences between *Fusobacterium*-high and *Fusobacterium*-low tumor samples (**Figures 5A–F**). Using cultured macrophages, Chen et al. showed that infection with *F. nucleatum* promoted M2 polarization of macrophages, and they proposed that the polarization is mediated by the IL6/STAT3/C-MYC signaling pathway via overexpression of *TLR4*, *STAT3*, and *c-myc* genes (27). Their results also indicated a significant increase in expression of *IL6* and *STAT3* in tumor biopsies. Our findings showed a significant increase in *IL6* gene expression, which correlated with the *Fusobacterium* load in the tumor biopsies (**Table 3**). In contrast, there was no notable increase in *STAT3* gene expression associated with *Fusobacterium*. Interestingly, we identified high expression of *SOCS3* in *Fusobacterium*-high samples, suggesting that JAK/STAT signaling is inhibited in this group. Furthermore, we examined expression of the c*-myc* and c*yclin D*1 genes in the tumor tissues. The expression of these genes in CRC tumors is mediated by the IL6/STAT3 signaling pathway (62). We examined the expression of these two genes as indicators for activation of IL6/STAT3 signaling, and our results showed no correlation between *Fusobacterium* abundance and higher expression of *c-myc* and c*yclin D*1 genes (**Figure 6**). Putting it together, our findings did not support *Fusobacterium-*associated M2 macrophage polarization *in vivo*, nor did they indicate that *Fusobacterium* promotes IL6/STAT3-mediated *c-myc* gene expression in tumor biopsies.

Another study proposed an S100A9-mediated mechanism for M2 polarization of macrophages (26). S100A9 is a pro-inflammatory mediator that has been shown to regulate myeloid-derived suppressor cells (MDSCs), which are a major component of the immunosuppressive TME (63). Our results showed that the expression of the *S100A9*, as well as *S100A8*, mRNAs were induced in *Fusobacterium*-high tumor samples (**Supplementary Table 3**). However, we did not find any correlation between expression of the *S100A9* gene and infiltration of M2 macrophages in the tumor biopsies in our cohort.

In summary, our results showed no indication for *Fusobacterium*-mediated M2 polarization of macrophages in the CRC tumor samples but, in contrast, a pro-inflammatory TME driven by inflammatory cytokines such as CXCL8, IL-6, and TNF-α. This discrepancy may reflect differences in sample type (*in vitro vs*. *in vivo*), tumor stage, or local microenvironmental signals. It should be noted that our study cohort mainly consisted of tumor biopsies taken under colonoscopy examination and represents tumors in their early stages, mostly Dukes stages A and B, while other studies showing M2 polarization of macrophages was conducted on tumor biopsies in later Dukes stages (**Table 1**) (26). Our results revealed a predominantly pro-inflammatory tumor microenvironment, driven by cytokines such as CXCL8, IL-6, and TNF-α, which create a favorable niche for tumor growth. Studies have shown that in later stages, the recruitment and polarization of M2 macrophages lead to the secretion of anti-inflammatory cytokines, including IL-10 and TGF-β (24). This results in an immunosuppressive tumor microenvironment that supports angiogenesis and facilitates tumor invasion and metastasis.

A strength of this study is the use of actual patient samples, which provides a more physiologically relevant perspective on tumor-microbiome interactions compared to *in vitro* models. However, the use of patient biopsies introduces variability that may obscure certain associations observed in controlled experimental settings. Additionally, our cohort size is limited, and transcriptional analyses alone may not capture post-translational modifications or protein-level interactions that influence immune responses. Future research should focus on expanding sample sizes to validate these findings and incorporate spatial transcriptomics to map *Fusobacterium*-immune interactions within the tumor microenvironment. Future functional studies using advanced patient-derived techniques to localize gene expression in tissue sections may help clarify *Fusobacterium’s* role in immune modulation.

## 5 Conclusion

Our findings align with previous research on how Fusobacterium influences the tumor microenvironment. According to our *in vivo* investigations, increased expression of several immune genes, such as *CXCL8*, *IL6*, *SPP1*, and *IDO1*, was associated with a high *Fusobacterium* load of several *Fusobacterium* species but dominated by *F. animalis*. The *fap2* gene was detected in most *F. animalis*- and *F. nucleatum*-positive samples and was actively transcribed, suggesting constitutive expression. Notably, tumors with high *Fusobacterium* abundance exhibited no significant change in M2 macrophage polarization.

## Data Availability

The original contributions presented in the study are included in the article/**Supplementary Material**. Further inquiries can be directed to the corresponding author.
